# *Romundina* and the evolutionary origin of teeth

**DOI:** 10.1098/rsbl.2015.0326

**Published:** 2015-06

**Authors:** Martin Rücklin, Philip C. J. Donoghue

**Affiliations:** 1 Naturalis Biodiversity Center, Postbus 9517, 2300 RA Leiden, The Netherlands; 2 School of Earth Sciences, University of Bristol, Life Sciences Building, Tyndall Avenue, Bristol BS8 1TQ, UK

**Keywords:** jawed vertebrates, placoderm, dental development, evolution, modularity

## Abstract

Theories on the origin of vertebrate teeth have long focused on chondrichthyans as reflecting a primitive condition—but this is better informed by the extinct placoderms, which constitute a sister clade or grade to the living gnathostomes. Here, we show that ‘supragnathal’ toothplates from the acanthothoracid placoderm *Romundina stellina* comprise multi-cuspid teeth, each composed of an enameloid cap and core of dentine. These were added sequentially, approximately circumferentially, about a pioneer tooth. Teeth are bound to a bony plate that grew with the addition of marginal teeth. Homologous toothplates in arthrodire placoderms exhibit a more ordered arrangement of teeth that lack enameloid, but their organization into a gnathal, bound by layers of cellular bone associated with the addition of each successional tooth, is the same. The presence of enameloid in the teeth of *Romundina* suggests that it has been lost in other placoderms. Its covariation in the teeth and dermal skeleton of placoderms suggests a lack of independence early in the evolution of jawed vertebrates. It also appears that the dentition—manifest as discrete gnathal ossifications—was developmentally discrete from the jaws during this formative episode of vertebrate evolution.

## 1. Introduction

Theories on the evolutionary origin of teeth have long been rooted in the condition manifest by chondrichthyans, as the most distant living outgroup to humans and because they exhibit a comparatively simple pattern of tooth replacement. However, their apparent simplicity is secondary given that the extinct placoderms, which constitute the sister lineage(s) to all other jawed vertebrates, exhibit a greater diversity and complexity of dentitions that better inform the nature of an ancestral gnathostome dentition. Dental development is best known in the arthrodiran placoderms, where teeth aggregrate to comprise gnathals ossified to the bony shaft of the lower jaw and the palatoquadrate [1]. This dentition is statodont; teeth were added successionally, replacing teeth that were not shed, bound together by an ossification associated with tooth addition [1]. However, arthrodires are derived regardless of whether placoderms are considered a clade or a grade [2,3] and the existence and nature of the dentition in other placoderm lineages are poorly known. Here, we describe the structure and growth of the supragnathal of *Romundina stellina*, a member of the acanthothoracid placoderms—considered an outgroup to a monophyletic Placodermi [4], or else an early branching lineage of paraphyletic ‘placoderms' [5]. As such, in comparison to other placoderms and crown-gnathostomes, *Romundina* might better inform the plesiomorphic nature of gnathostome dentitions. We used synchrotron radiation X-ray tomographic microscopy (SRXTM) to obtain a high-resolution volumetric characterization of gnathals from *Romundina* and, for comparison, the arthrodire *Compagopiscis croucheri.* We subjected these datasets to computed tomographic analysis to elucidate the structure and infer the development of these skeletal structures.

## 2. Material and methods

The supragnathal and associated skeletal elements are from acid-insoluble residues associated with the holotype of *R. stellina*, from the Early Devonian (Lochkovian) of Prince of Wales Island, Canada [6], housed in the Naturhistoriska Riksmuseet, Stockholm (NRM-PZ). For comparison, we studied posterior supragnathals of *C. croucheri* from the Upper Devonian, Frasnian, Gogo Formation of Australia, reposited at the Natural History Museum London (NHMUK PV). Volumetric characterization of the specimens was achieved using SRXTM [7] at the TOMCAT (X02DA) beamline of the Swiss Light Source, Paul Scherrer Institut, Switzerland (voxel dimensions 0.74 and 1.85 µm) and a SkyScan 1172 XTM at the University of Bristol (voxel dimensions 10 µm); the raw slice data are available at http://dx.doi.org/10.5523/bris.7h9gynbsui4u1hap471inrlua and as movie files in the electronic supplementary material. These data were analysed using AVIZO 8.01 (www.fei.com).

## 3. Results

**Figure 1.**
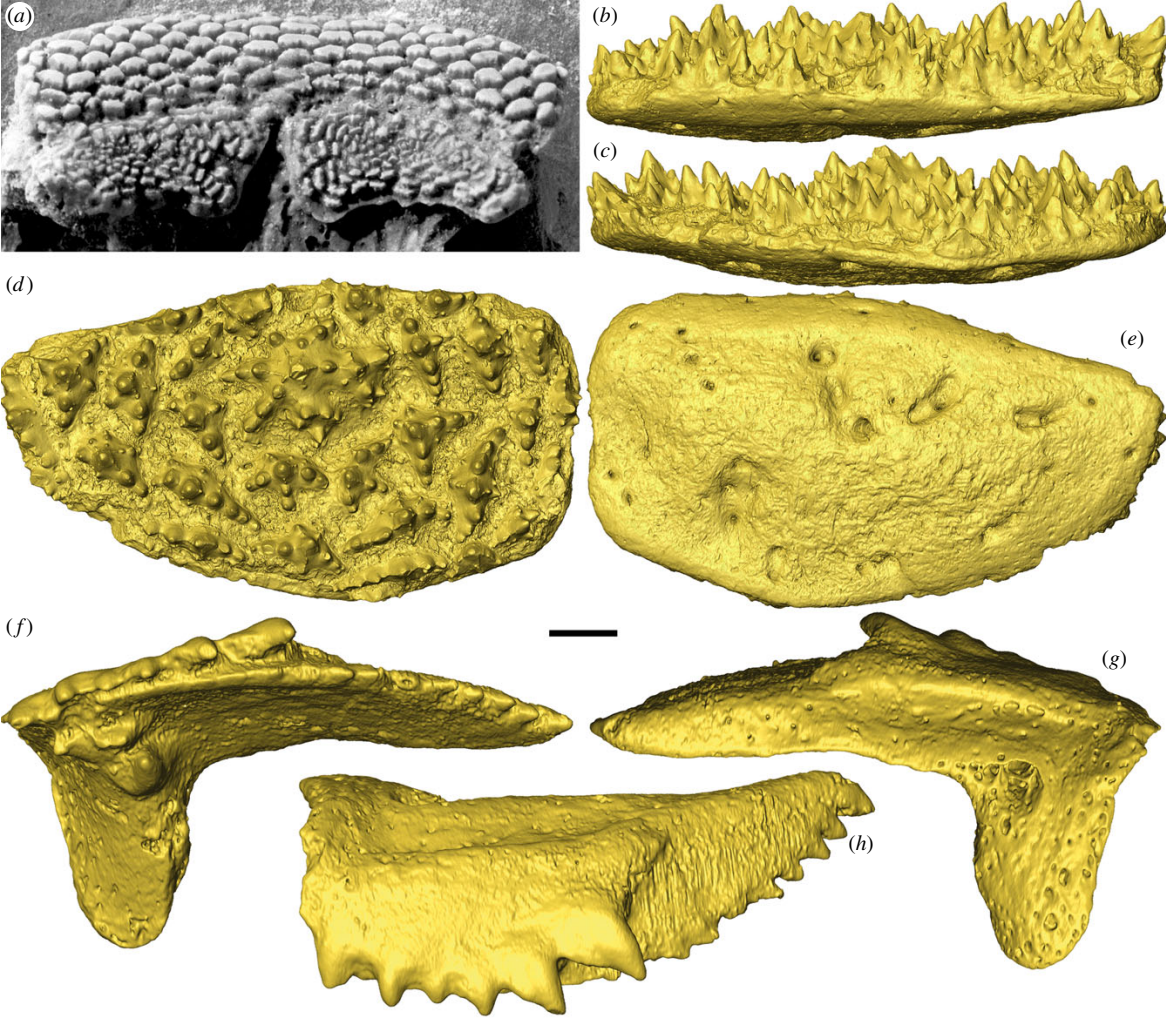
Acanthothoracid placoderm (same specimen as in [4]) and surface renderings (gold) of *Romundina stellina* and *Compagopiscis croucheri*. Upper dental plates (anterior supragnathals, ASG) in occlusal view (*a*). Right ASG of *R. stellina* (NRM-PZ P.15956) based on SRXTM data (*b–e*). (*b*) Distal, (*c*) proximal, (*d*) occlusal and (*e*) dorsal views. Left posterior supragnathal (PSG) of *C. croucheri* (NHMUK PV P.50943), based on MicroCT data (*f–h*). (*f*) Occlusal, (*g*) dorsal and (*h*) distal views. Scale bar represents 1.68 mm in (*a*), 178 µm in (*b*–*e*) and 206 µm in (*f*–*h*).

**Figure 2.**
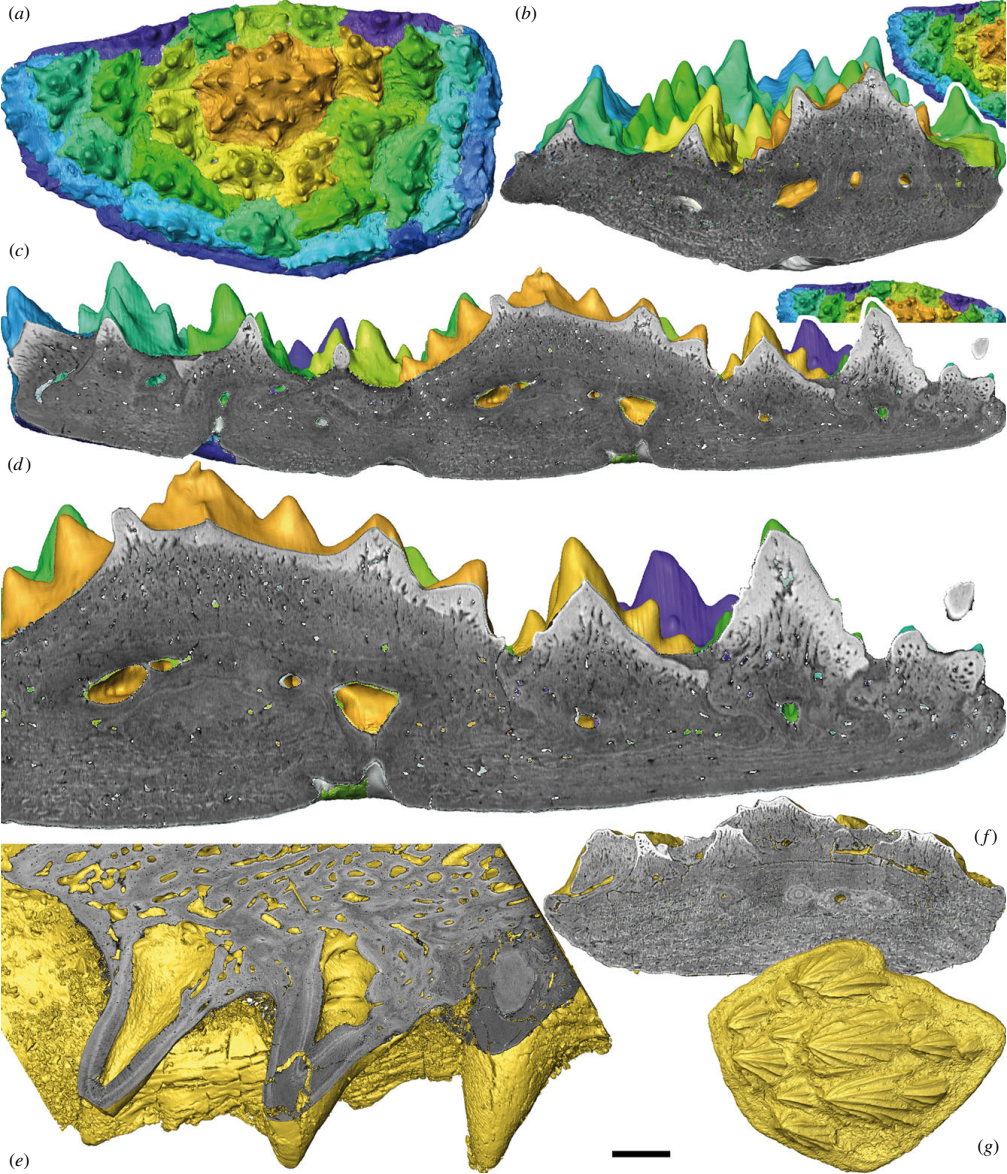
Segmentation and virtual sections of SRXTM characterizations of a *Romundina stellina* supragnathal (NRM-PZ P.15956), dermal scale (NRM-PZ P.15952) and *Compagopiscis croucheri* supragnathal (NHMUK PV P.57629). (*a–d*) Right ASG of *R. stellina*. (*a*) Segmented sclerochronology of the dental plate following lines of arrested growth. Colour scheme (from gold to purple) represents the sequence of tooth addition. (*b*) Transverse and (*c*) longitudinal virtual sections showing addition of teeth and basal layer. (*d*) Detail of (*c*) showing enameloid/semidentine border and Sharpey's fibres. (*e*) Detailed virtual section of the right PSG of *C. croucheri*. (*f*) Virtual section and (*g*) dorsal view of the dermal scale of *R. stellina*. Scale bar represents 180 µm in (*a*), 97 µm in (*b*), 86 µm in (*c*), 50 µm in (*d*), 157 µm in (*e*), 96 µm in (*f*) and 224 µm in (*g*).

Only the upper dental plates (supragnathals) are known for *Romundina*, described from the palatal surface of an endocranium as ‘a pair of symmetrical flat plates with a specific ornament combining radiating and concentric rows with a centrifugal growth’ [4, p. 114]. The upper dental plates are flat and oval-shaped with an ornament of multi-cuspid tubercles (figure 1*a*). The new material is identified as a gnathal plate of *Romundina* on grounds of equivalent size and similar shape, and its derivation in association with the holotype of *R. stellina* [6]. The gnathal has a prominent central tubercle with a central cusp from which six radial ridges extend, each bearing a series of aligned cusps. This is surrounded by smaller tubercles, each exhibiting the same basic arrangement of cusps, though one or more of the radial ridges may not be developed. Thus, marginal tubercles exhibit elongate ridges aligned with the circumference of the gnathal plate (figures 1*a,d* and 2*a*).

Tomographic sections reveal that the gnathal plate comprises three layers: a superficial layer composed of tubercles, a medial vascular layer and a basal lamellar layer (figure 2*b–d*). The tubercles generally lack a coherent vascular cavity, but they comprise dentine with odontoblast lacunae, characteristic of semidentine, that converge on local chambers associated with the middle vascular layer. The dentine exhibits an irregular boundary with an outer hypermineralized capping layer of enameloid that is continuous between component cusps of each tubercle and permeated by the odontoblast canaliculi (figure 2*b–d*). Inner areas of dentine tissues with canaliculi and cell lacunae are characteristic for semidentine (figure 2*d*). The vascular middle layer is dominated by canals that extend through the basal and the superficial layers, opening between tubercles. The basal layer consists of lamellar bone that is generally organized into a plywood-like structure characteristic of isopedin, though it comprises fibre bundles that are approximately circular in cross-section, akin to the osteostracan and galeaspid dermal skeletons, rather than the sheet-like organization seen in actinopterygians [8]. This structure is permeated by Sharpey's fibres centrally (figure 2*b–d*) and disintegrates locally into spheritic mineralization characteristic of rapid growth or the absence of a coherent collagen matrix [9].

The tomographic data also reveal clearly that the tubercles were added episodically to the margins of the gnathal plate, evidenced by growth arrest lines that occur between tubercles that developed on the margins of older, earlier formed, tubercles (figure 2*b–d*). These growth lines can be traced continuing through the middle and basal layers, demonstrating that the bony plate grew in width and thickness in association with the addition of tubercles at the margins. Tracing these growth lines digitally revealed that the tubercles were added marginally in concert (figure 2*b–d*). Thus, the tubercles were added radially in respect of the pioneer, but restricted by the distal limit of the oral cavity where the gnathal plates abutted the premedian plate, as may be inferred in comparison to supragnathal plates *in situ* (figure 1*a*).

The supragnathal plates of the brachythoracid arthrodire *C. croucheri* occur bilaterally as separate anterior and posterior elements; either the anterior element is homologous to the single bilateral supragnathal plates in *Romundina* or else they are perhaps collectively equivalent. They each have a central cusp, from which branch three rows, only two of which comprise more than a few cusps (figure 1*f–h*). Tomographic sections reveal that each cusp has a distinct and voluminous pulp cavity (figure 2*e*). Growth arrest lines indicate that the cusps were added in succession, to the margin of the next oldest cusp within the row, and relative age of the cusps is also reflected in the degree to which the pulp cavities are centripetally infilled by dentine. The addition of each cusp is continuous with the bone added to the basal plate uniting the component cusps. The growth lines become indistinct where remodelling of the vasculature has occurred (figure 2*e*).

## 4. Discussion

The surface morphology of the tubercles comprising the supragnathal in *Romundina* is quite distinct from the morphology of the dermal tubercles, though they have a common composition. Given their statodont pattern of replacement, with new tubercles added at the margins of the oral surface, their toothlike composition, and the homology of the gnathals to the supragnathals of arthrodires such as *Compagopiscis*, which have already been interpreted as teeth [1], we interpret the supragnathals of *Romundina* as comprising teeth. Nevertheless, the structure and composition of the supragnathal toothplate in *Romundina* is surprising given what has been known previously concerning the structure of placoderm dental elements. The simple radial organization of the tubercles is similar to the compound oral denticles in the jawless thelodont *Loganellia* [10], but it is quite distinct from the strictly ordered arrangement of the tubercles comprising the gnathals of arthrodire placoderms, including the supragnathals described here from *Compagopiscis*, where the tubercles are monocuspid and arranged along discrete vectors [1]. Conversely, the multicuspid gnathal tubercles in *Romundina* are considerably more complex. These differences are perhaps reflected in the differing degrees of gnathal occlusion, where the supra- and infra-gnathals of *Compagopiscis* should be envisaged to occlude with precision, whereas it is difficult to conceive any meaningful degree of occlusion in *Romundina,* perhaps because it would be precluded by the complex interdigitating, space-filling morphology of the tubercles comprising the functional surface of the gnathals. Thus, these differences may as equally reflect poorer constraint of jaw articulation, as of dental development, in the earliest jawed vertebrates.

The presence of an enameloid capping layer to the teeth of *Romundina* is not unusual in comparison to the composition of osteichthyan and chondrichthyan teeth; however, it contrasts with the structure of teeth in arthrodires, which have been shown to lack enameloid [1]. Enameloid is also present in the external dermal tubercles of *Romundina* (figure 2*f,g*), which is a primitive feature for ostracoderms [9], but it is unusual for most placoderms where enameloid is absent through loss [11]. This suggests that the absence of enameloid from the teeth of arthrodires [1] is also a consequence of loss and that the teeth of the earliest jawed vertebrates included a hypermineralized capping layer of enameloid.

Indeed, the coordinated presence versus absence of enameloid associated with the dermal and oral odontodes may be more illuminating, suggestive of the non-independence of these skeletal systems in the earliest jawed vertebrates. This view is entirely compatible with the view that teeth evolved through the extension of odontogenic competence from external to internal epithelia, but incompatible with the view that internal and external odontodes evolved independently from a non-skeletal antecedent organ system [12].

In either instance, the organization of teeth into gnathals that occur distinct from other aspects of the dermal and endoskeletal systems appears to be widespread among placoderms, including acanthothoracids and arthrodires. As such, this may reflect a primitive condition for jawed vertebrates, and the intimate association of teeth and jaws may be an entirely derived feature of osteichthyans.

## 5. Conclusion

The gnathals of *Romundina* may reflect a primitive condition for placoderms and, indeed, jawed vertebrates more generally: discrete developmental units that comprise teeth composed of dentine and capped with enameloid. As such, the search for the origin of teeth must be extended deeper into gnathostome phylogeny. However, the organization of teeth and their intimate developmental association with jaws appear to be derived phenomena that evolved later in jawed vertebrate phylogeny.

## Supplementary Material

Electronic Supplementary Information text

## Data accessibility

Data used in this manuscript are archived at http://dx.doi.org/10.5523/bris.7h9gynbsui4u1hap471inrlua and as movie files in electronic supplementary material.
