# Supplementary material for: Romundina and the evolutionary origin of teeth
Source: Biol Lett. 2015 Jun;11(6):20150326. doi: 10.1098/rsbl.2015.0326 (PMC4528481; doi:10.1098/rsbl.2015.0326)
Supplement: Electronic Supplementary Information text [file rsbl20150326supp1.doc]

# **Eletronic Supplementary Information**

The supragnathal and associated skeletal elements of *Romundina stellina* were recovered from samples associated with the holotype from the Early Devonian (Lochkovian) of Prince of Wales Island, Canada , housed in the Naturhistoriska Riksmuseet, Stockholm (NRM-PZ). The specimens were recovered from acid-insoluble residues of limestone, dissolved with dilute acetic acid by Tor Ørvig (NRM-PZ) and with 10% buffered acetic acid by the authors. The samples otherwise contain vertebrate microremains and mesoremains of pteraspid and tessellate heterostracans, as well as acanthodians, thelodonts and conodonts.

# The dermal and oral skeletal elements of *Romundina* can be distinguished from all others on the basis that the denticles are composed in part of semidentine – a tissue that is peculiar to placoderm-grade vertebrates . The dermal and oral denticles have enameloid caps; this is extremely unusual for placoderms, but it has been described previously as a feature of *Romundina* . The shape and arrangement of the oral denticles is identical to those described previously in situ in *Romundina* . We can exclude the possibility that these represent dermal elements of *Romundina* since the dermal odontodes in this taxon exhibit a distinct striated ornament not seen in the oral odontodes described by us or by others .

The reconstructed raw slice data are available at: <http://dx.doi.org/10.5523/bris.7h9gynbsui4u1hap471inrlua>.

Data consist of four folders each includes reconstructed tiff files:

The supragnathal of *Romundina stellina* (NRM-PZ P.15956; Fig. 1b-e, Fig. 2a-d), as two blocks of a stacked scan, folders are named “MR07A_B1_” and “MR07A_B2_”. The specimen was scanned with a X10 objective resulting voxel size is 0.74 µm. Energy of 13 keV with 1501 projections equi-angularly distributed over 180˚ and exposure time of 175 ms was used.

The dermal scale of *Romundina stellina* (NRM-PZ P.15952; Fig. 2f, e), reconstructed tiffs in folder “MR014a” were scanned with X10 objective resulting voxel size of 0.74 µm. Energy of 14 keV with 1501 projections and exposure time of 175 ms was used.

The posterior supragnathal of *Compagopiscis croucheri* (NHMUK PV P.57629, Fig. 2e), reconstructed tiffs in folder “MR13A” were scanned with a X4 objective resulting voxel size of 1.85 µm. Energy of 20 keV with 1501 projections and exposure time of 150 ms.

Three video files of the slice data are included as mpg files:

Video 1. Reconstructed slice data of the supragnathal of *Romundina stellina* (NRM-PZ P.15956; Fig. 1b-e, Fig. 2a-d). The video follows transverse slices from lateral to medial, generated with AVIZO 8.1.

Video 2. Reconstructed slice data of the dermal scale of *Romundina stellina* (NRM-PZ P.15952; Fig. 2f, e). The video shows transverse slices generated with AVIZO 8.1.

Video 3. Reconstructed slice data of the central part of the supragnathal of *Compagopiscis croucheri* (NHMUK PV P.57629, Fig. 2e). The video follows longitudinal slices from distal to proximal, generated with AVIZO 8.1.

1. Ørvig T. 1975 Description, with special reference to the dermal skeleton, of a new radotinid arthrodire from the Gedinnian of Arctic Canada. *In* Problemes actuels de Paléontologie - Évolution des vertébrés. *Colloque international CNRS* **218**, 41-71.

2. Young G.C. 2010 Placoderms (armored fish): dominant vertebrates of the Devonian Period. *Annual Review of Earth and Planetary Sciences* **38**(1), 523-550. (doi:doi:10.1146/annurev-earth-040809-152507).

3. Giles S., Rücklin M., Donoghue P.C.J. 2013 Histology of "placoderm" dermal skeletons: Implications for the nature of the ancestral gnathostome. *Journal of Morphology* **274**, 627-644.

4. Goujet D., Young G.C. 2004 Placoderm anatomy and phylogeny: new insights. In *Recent advances in the origin and early radiation of vertebrates* (eds. Arratia G., Wilson M.V.H., Cloutier R.), pp. 109-126. München, Pfeil.
